# Long non-coding RNA LINC01116 acts as an oncogene in prostate cancer cells through regulation of miR-744-5p/UBE2L3 axis

**DOI:** 10.1186/s12935-021-01843-w

**Published:** 2021-03-16

**Authors:** Shengjie Yu, Huihong Yu, Yuanfeng Zhang, Chuan Liu, Weili Zhang, Yunyun Zhang

**Affiliations:** 1 Department of Urology, The Second Affiliated Hospital, Chongqing Medical University, No. 76 Linjiang Road, Chongqing, 400016 China; 2 Department of Gastroenterology, The Second Affiliated Hospital, Chongqing Medical University, Chongqing, 400016 China; 3 Tumor Radiotherapy Center, Cancer Hospital Affiliated to Chongqing University, No. 181 Hanyu Road, Chongqing, 400030 China

**Keywords:** LINC01116, miR-744-5p, UBE2L3, Prostate cancer

## Abstract

**Background:**

Long non-coding RNA (lncRNA) has been confirmed to exert a critical effect on the progression of tumors, including prostate cancer. Previous literature has demonstrated LINC01116 involves in activities of multiple cancers. However, the underlying role of LINC01116 in prostate cancer remains unclear.

**Methods:**

qRT-PCR measured the expression of LINC01116 in prostate cancer cells. EdU experiment was used to detect cell proliferation. Transwell assays detected cell migration and invasion. Immunofluorescence staining and western blot assays were utilized to measure EMT progress. The binding relationship between RNAs was confirmed by a series of mechanism assays. In addition, rescue experiments were conducted to verify the relationship among RNAs.

**Results:**

LINC01116 was found to be highly expressed in prostate cancer cells. Functional assays indicated that inhibition of LINC01116 could suppress cell proliferation, migration, invasion and EMT progress. Also, miR-744-5p was proven to bind with LINC01116. Moreover, UBE2L3 was verified as the target gene of miR-744-5p. In rescue assays, we discovered that inhibited miR-744-5p or overexpressed UBE2L3 could offset the suppressive influence of silencing LINC01116 on prostate cancer cells.

**Conclusion:**

Our study suggested that lncRNA LINC01116 acted as an oncogene in prostate cancer and accelerated prostate cancer cell growth through regulating miR-744-5p/UBE2L3 axis.

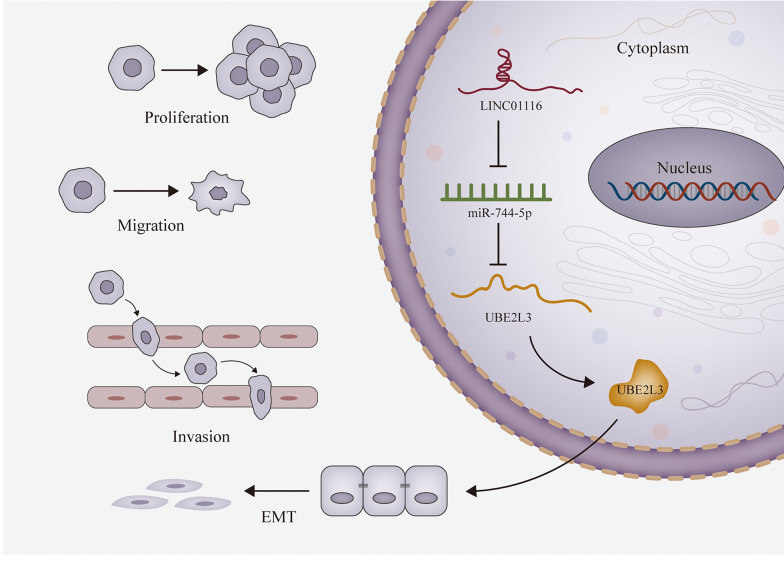

## Background

Statistics have showed that prostate cancer is confirmed to be the second prevalent cancer in male all over the world [1]. Detection of prostate cancer has been advanced with the development of prostate specific antigen (PSA) screening, magnetic resonance imaging (MRI) and new prostate biopsy methods [2]. Although the prognosis of patients with localized prostate cancer has been improved by radical prostatectomy and radiotherapy, currently available treatments have limited efficacy [3, 4]. Thus, it is of great interest to delve into the potential mechanism of prostate cancer and seek out novel perspective to better understand this cancer.

Long non-coding RNA (lncRNA) is the member of non-coding RNA family longer than 200 nt [5]. In several studies, the mechanisms of lncRNA in a variety of cancers were revealed. Increasing evidence showed that changes in lncRNA expression contributed to cell proliferation and transformation in malignant tumors including prostate cancer [6]. The significant role of lncRNAs in regulating the development of human cancer has been focused recently [7–9]. Salmena’s competitive endogenous RNA (ceRNA) theory provides new insights into the mechanism of lncRNA and its role in cancer [10]. Long intergenic non-protein coding RNA 1116 (LINC01116) was found to be linked to overall survival rate of patients with breast cancer [11]. Also, LINC01116 enhanced the progression of epithelial ovarian cancer through regulating cell growth and development [12]. However, whether LINC01116 could function in prostate cancer is still not determined.

The main focus of the present study was to delve into the impact and potential mechanism of LIINC01116 in prostate cancer cells. Our study results manifested that LINC01116 presented abnormal expression in cancer cells. Silencing LINC01116 could inhibit the growth of cancer cells, as well as EMT process, by regulating miR-744-5p/ubiquitin conjugating enzyme E2 L3 (UBE2L3) axis. The study might provide a novel perspective in understanding prostate cancer.

## Methods

### Cell culture

Human prostate cancer cell lines (DU145, PC3, LNCAP, 22RV1, VCaP) and human prostate epithelial cell line (RWPE-1) were acquired from American Type Culture Collection (ATCC; Manassas, VA, USA) for study. RWPE-1 cells were cultivated in Keratinocyte Serum Free Medium (K-SFM; Gibco, Grand Island, NY, USA) in 5% CO_2_ at 37 °C. As for prostate cancer cell lines, LNCAP and 22RV1 were routinely cultured in RPMI-1640 medium (Thermo Fisher Scientific, Inc., Waltham, MA, USA) in 5% CO_2_ at 37 °C with 10% fetal bovine serum (FBS; Gibco, Grand Island, NY, USA), 100 mg/ml streptomycin and 100 U/ml penicillin as the medium supplements. Under the same culture conditions, DU145 cells were cultivated in Eagle’s Minimum Essential Medium; PC3 cells were cultivated in F-12K medium; VCaP cells were cultivated in Dulbecco’s Modified Eagle’s Medium (Gibco).

### Quantitative real-time polymerase chain reaction (qRT-PCR)

Total RNA extraction was first completed by use of the TRIzol Reagent (Invitrogen, Carlsbad, CA, USA), and then 1 μg of total RNA was used for generating cDNA with PrimeScript Reverse Transcriptase Kit (Takara, Shiga, Japan). The determination of gene expression was conducted by PCR with SYBR Green PCR Kit (Takara). Experimental results were then calculated based on 2^−ΔΔCt^ method and standardized to U6 snRNA or GAPDH mRNA. Each independent experiment was carried out at least thrice.

### Plasmid transfection

The full-length cDNA sequence of UBE2L3 was acquired and inserted into pcDNA3.1 expressing vector (Invitrogen) for overexpressing UBE2L3, with empty vector as control. The specific shRNAs to LINC01116 (sh-LINC01116#1/2) or UBE2L3 (sh-UBE2L3#1/2) and their relative negative control (sh-NC) were constructed by GenePharma (Shanghai, China). The miR-744-5p mimics and NC mimics were constructed by Ribobio (Guangzhou, China), as well as miR-744-5p inhibitor and NC inhibitor. All these were transfected for 48 h into DU145 and LNCAP, by means of Lipofectamine 3000 (Invitrogen). Each independent experiment was carried out at least thrice.

### Western blot

At first, RIPA buffer was utilized to obtain the protein lysates. Total protein was extracted and the proteins were then treated with SDS-PAGE and transferred to the PVDF membranes. Subsequently, membranes blocked by 5% skimmed milk were co-cultivated with specific primary antibodies against E-cadherin, N-cadherin, Vimentin, ZEB1, Slug and Twist overnight at 4 °C. After that, secondary antibodies were added for cultivation. Eventually, the proteins were measured via enhanced chemiluminescence (ECL) detection system. Each independent experiment was carried out at least thrice.

### 5-Ethynyl-2′-deoxyuridine (EdU) assay

By use of the BeyoClick™ EdU Cell Proliferation Kit (Beyotime, Shanghai, China), EdU assay was implemented in DU145 and LNCAP as instructed by provider. After transfection, cells (1 × 10^5^ per well) were seeded into 6-well plates before being washed with PBS, and then EdU medium was added into cells for 2 h. Next, 4% paraformaldehyde (PFA) was added for 15-min fixing. Cell nucleus was visualized via 4′,6-diamidino-2-phenylindole (DAPI) solution (Beyotime) using fluorescence microscope (Olympus, Tokyo, Japan). Each independent experiment was carried out at least thrice.

### Transwell assay

Cell invasion was studied using the 24-well transwell inserts coated with 30 μg of Matrigel (BD Biosciences, Franklin Lakes, NJ, USA). Cell migration was detected similarly, without Matrigel. Transfected DU145 and LNCAP cells (2 × 10^4^ per well) were cultured in the top compartment without serum, with lower compartment supplemented with 10% FBS. After 24-h incubation in 5% CO_2_, cells remaining on the top compartment were cleared and cells moving to the lower compartment were fixed by 4% PFA. After that, invaded or migratory cells were dyed by 0.5% crystal violet before observation under optical microscope (Olympus). Number of invaded or migrated cells in 5 randomly selected fields was counted. Each independent experiment was carried out at least thrice.

### Immunofluorescence staining (IF)

Transfected DU145 and LNCAP cells on coverslips were first fixed by 4% PFA for 10 min, and then blocked by 5% BSA for 30 min. Next, cells were cultured with primary antibodies targeting E-cadherin and N-cadherin for a whole night at 4 °C, and then fluorescence-conjugated secondary antibodies were added and co-cultured at room temperature. After DAPI staining for nuclear detection, fluorescence analysis was conducted with Olympus. Each independent experiment was carried out at least thrice.

### Fluorescence in situ hybridization (FISH)

DU145 and LNCAP cells were fixed by 4% PFA for 15 min, digested by pepsin and dehydrated by ethanol. After that, the air-dried cells were cultured with the specific FISH probe to LINC01116 in hybridization buffer. Cell samples were treated in Hoechst staining solution for detection of cell nucleus. Samples were visualized for analysis with Olympus fluorescence microscope. Each independent experiment was carried out at least thrice.

### Nucleus-cytoplasm separation assay

Using PARIS™ Kit (Invitrogen), nucleus-cytoplasm separation assay was undertaken. 1 × 10^6^ cell samples of DU145 and LNCAP were prepared in precooled PBS, and then lysed with cell fractionation buffer. After centrifugation, cell disruption buffer was added. LINC01116 content was examined by qRT-PCR. GAPDH and U6 served as the cytoplasmic control and nuclear control, respectively. Each independent experiment was carried out at least thrice.

### RNA pull down

The miR-744-5p sequences which contained the wild-type (WT) and mutated (Mut) binding sites of LINC01116 or UBE2L3 were biotin-labeled to construct Bio-miR-744-5p-WT/Mut probes. After that, cell protein extracts were mixed with Bio-miR-744-5p-WT/Mut or Bio-NC probes, followed by adding magnetic beads for 1 h. The mixture pulled down was finally assessed using qRT-PCR. Each independent experiment was carried out at least thrice.

### Luciferase reporter assay

The LINC01116 or UBE2L3 fragments covering miR-744-5p wild-type and mutant binding sites were prepared for luciferase analysis using the pmirGLO reporter vectors (Promega, Madison, WI, USA). After that, the constructs were co-transfected with miR-744-5p mimics or NC mimics into DU145 and LNCAP cells. 48 h later, the detection of luciferase activity was achieved using luciferase reporter assay system (Promega). Each independent experiment was carried out at least thrice.

### RNA binding protein immunoprecipitation (RIP)

RIP assay was implemented in DU145 and LNCAP cells (1 × 10^6^) by use of Magna RIP™ RNA-Binding Protein Immunoprecipitation Kit (Millipore, Bedford, MA, USA). The human anti-Ago2 and normal mouse anti-IgG antibodies were utilized. The cultured cells were incubated with RIP buffer which contained antibody-bound magnetic beads. After that, the immunoprecipitated RNAs were extracted and then purified for qRT-PCR analysis. Each independent experiment was carried out at least thrice.

### Statistical analyses

The procedures of each assay were conducted at least three times. Experimental data were given as the mean ± standard deviation (SD). Student’s t-test or one-way analysis of variance (ANOVA) was employed for comparisons of groups and GraphPad PRISM 6 (GraphPad, San Diego, CA, USA) was utilized to draw pictures. And p value less than 0.05 was inferred to be statistically significant.

## Results

### LINC01116 is highly expressed in prostate cancer cells and promotes cell growth

**Fig. 1:**
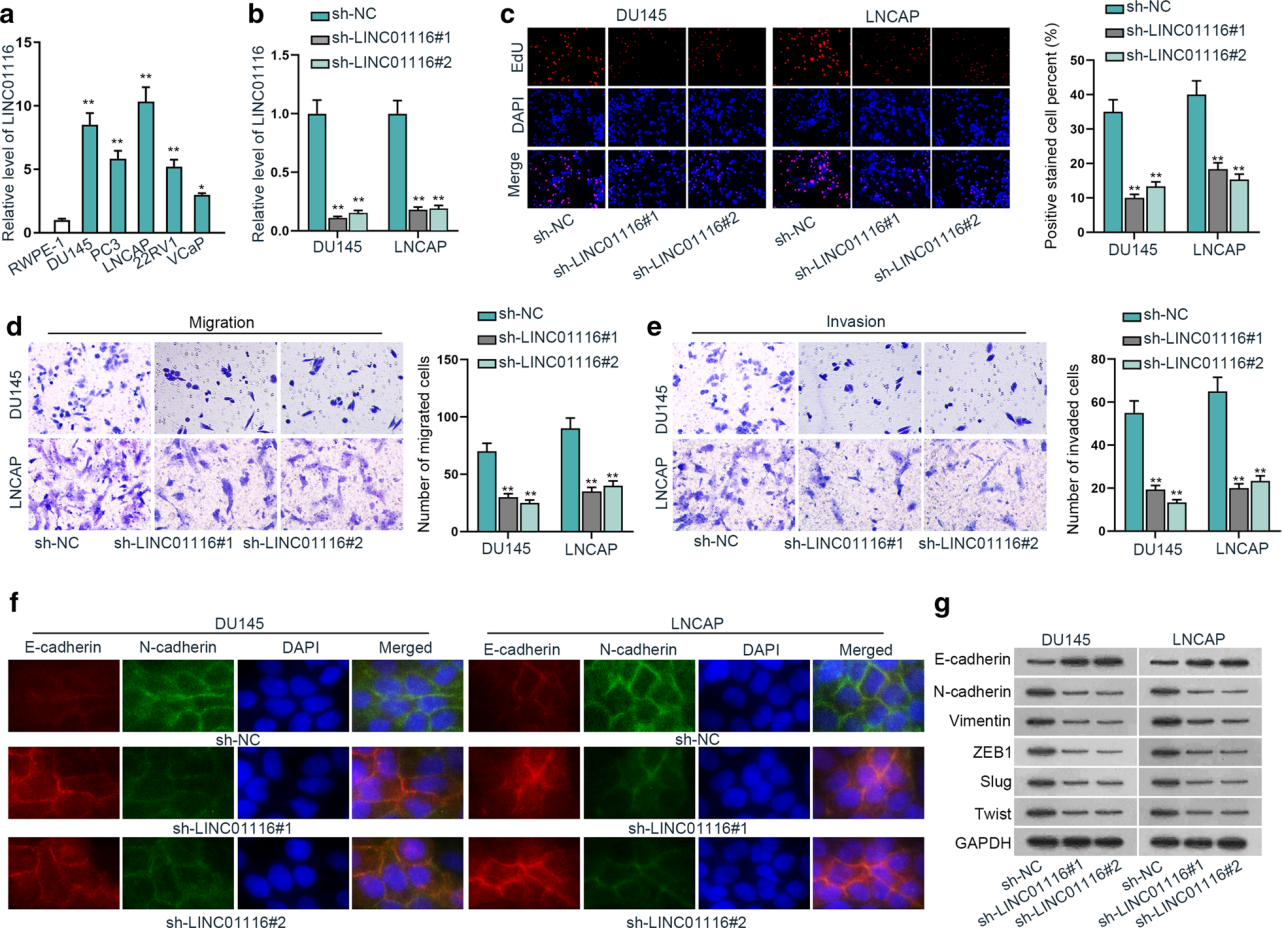
LINC01116 is highly expressed in prostate cancer cells and promotes cell growth. **a** qRT-PCR results showed that LINC01116 was at a higher stage of expression in DU145 and LNCAP. **b** qRT-PCR tested the expression level of LINC01116 when cells were transfected with sh-LINC01116. **c** EdU assay was executed to observe the cell proliferative capability. **d**, **e** Transwell assay detected cell migration and invasion. **f** IF staining assay measured the EMT progress. **g** Expression of six EMT markers was quantified by means of western blot. *P < 0.05, **P < 0.01

For the detection of LINC01116 function in prostate cancer, we firstly measured the expression of LINC01116 in prostate cancer cells. The qRT-PCR results showed that LINC01116 was highly expressed in five cancer cells (DU145, PC3, LNCAP, 22RV1 and VCaP) compared with the normal cell line (RWPE-1) (Fig. 1a). Meanwhile, LINC01116 was found at a higher stage of expression in DU145 and LNCAP, so we took DU145 and LNCAP as experimental cells. It was later found that transfection of sh-LINC01116 into cells could inhibit the expression level of LINC01116 (Fig. 1b). Then EdU results implied that silencing LINC01116 led to suppressed cell proliferative ability (Fig. 1c). Then transwell assay was conducted to detect cell migratory and invasive capabilities, and we detected that inhibiting LINC01116 would hamper the migration and invasion activities of the cancer cells (Fig. 1d and Additional file 1: Fig. S1E). Additionally, IF staining results reflected down-regulating the expression of LINC01116 could restrain EMT process (Fig. 1f and Additional file 1: Fig. S1A). To further validate the influence of LINC01116 inhibition on EMT process, we utilized qRT-PCR and western blot to measure expression of certain EMT-related proteins (Fig. 1g and Additional file 1: Fig. S1B), and according to the results, LINC01116 silence resulted in inhibited EMT process. Therefore, LINC01116 could promote cell proliferative, migratory, invasive and EMT progress of prostate cancer cells.

### LINC01116 sponges miR-744-5p in prostate cancer cells

**Fig. 2:**
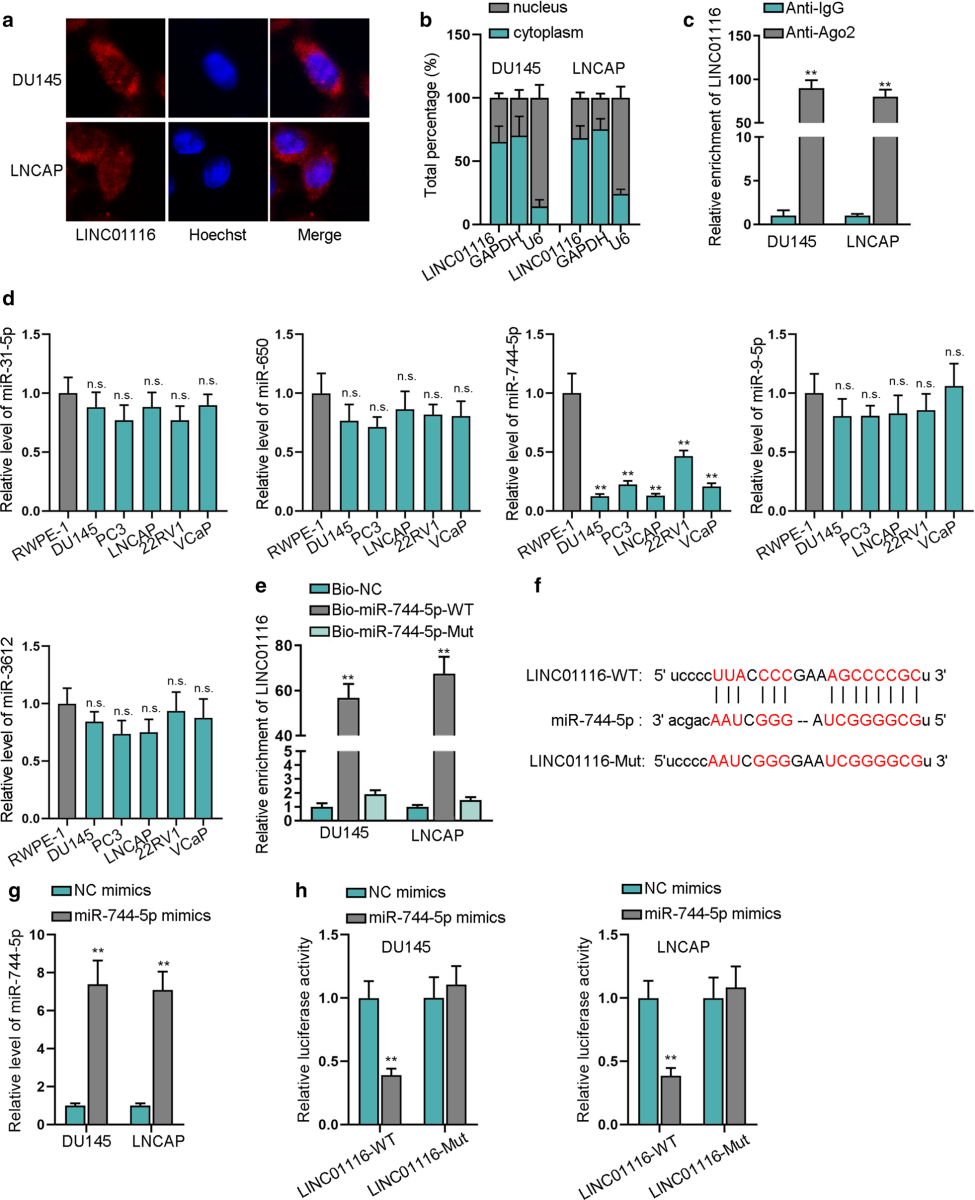
LINC01116 sponges miR-744-5p in prostate cancer cells. **a**, **b** FISH assay and nucleus-cytoplasm separation assay were utilized to determine the location of LINC01116. **c** RIP assay was implemented to assess the relationship of LINC01116 and Ago2. **d** The expression level of the five possible miRNAs was measured via qRT-PCR. **e** RNA pull down assay was conducted to detect the correlation of LINC01116 and miR-744-5p. **f** The alignment of LINC01116 and miR-744-5p were predicted by ENCORI. **g** The effectiveness of miR-744-5p mimics was assessed through qRT-PCR. **h** Luciferase reporter experiment was carried out for determining the binding relation of LINC01116 and miR-744-5p. **P < 0.01, n.s.: no significance

First, from FISH assay and nucleus-cytoplasm separation assay, we found LINC01116 was primarily located at cytoplasm (Fig. 2a and b). Then RIP assay was conducted and we discovered that LINC01116 could be enriched by Anti-Ago2, manifesting LINC01116 might function as a ceRNA to sponge miRNAs (Fig. 2c). We screened out miR-744-5p and other four miRNAs for further assays by using ENCORI (http://starbase.sysu.edu.cn/index.php) database (Additional file 1: Fig. S1C). Later, we executed qRT-PCR analysis for further screening and its results manifested that the expression level of miR-744-5p was abnormally low in the five cancer cells in comparison with other miRNAs (Fig. 2d). Thus, miR-744-5p was considered to combine with LINC01116. For verification, we carried out pull down assay and found that LINC01116 could be enriched by biotinylated miR-744-5p-WT (Fig. 2e). Afterwards, we mutated the binding site between LINC01116 and miR-744-5p (Fig. 2f). And based on qRT-PCR analysis, after transfection of miR-744-5p mimics into DU145 and LNCAP, miR-744-5p expression was increased (Fig. 2g). After that, luciferase reporter experiment proved that overexpressed miR-744-5p contributed to reduced relative luciferase activity of LINC01116-WT, while that of LINC01116-Mut was hardly affected by overexpressed miR-744-5p (Fig. 2h). Therefore, it was proved that LINC01116 could bind to miR-744-5p in prostate cancer cells.

### Upregulated miR-744-5p represses the cell growth of prostate cancer

**Fig. 3:**
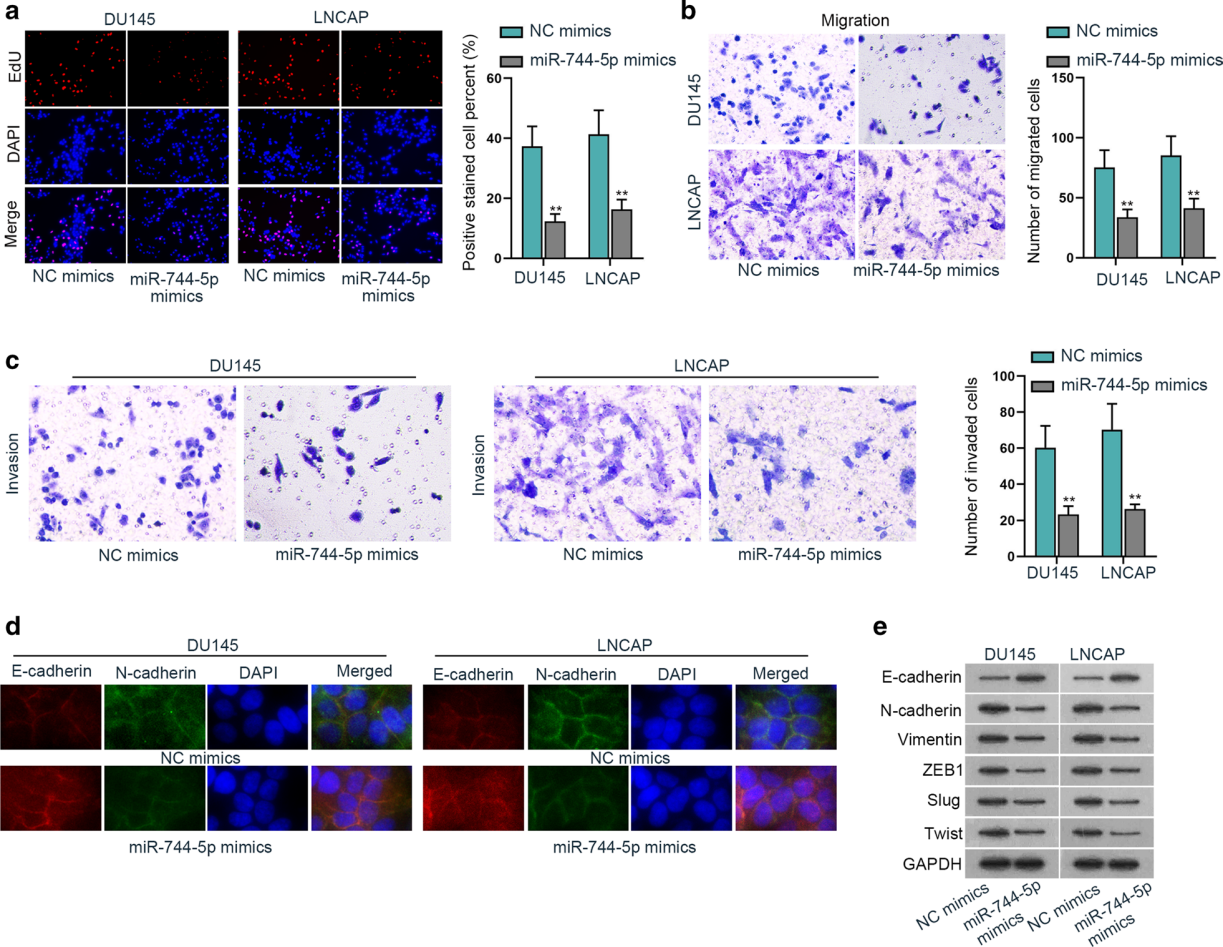
Upregulated miR-744-5p represses the cell growth of prostate cancer. **a** EdU assay was utilized to detect cell proliferation when miR-744-5p was overexpressed in cells. **b**, **c** Transwell assays measured cell migratory and invasive abilities after overexpressing miR-744-5p. **d** IF staining assay was applied for estimating the influence of upregulating miR-744-5p on EMT progress. **e** Expression of E-cadherin and other five EMT-related proteins was quantified by means of western blot. **P < 0.01

First, EdU assay results reflected that after transfection with miR-744-5p mimics, the proliferation capacity of DU145 and LNCAP cells was inhibited (Fig. 3a). Later, transwell assays were executed to assess the influence of overexpressed miR-744-5p on cell migrated and invasive capabilities, and the results proved that upregulated miR-744-5p could decrease the amount of migratory and invaded cells, suggesting cell migration and invasion were suppressed (Fig. 3b and c). Additionally, IF staining assay showed that transfection of miR-744-5p mimics would inhibit the EMT process of cancer cells (Fig. 3d and Additional file 1: S1D). For further validation of miR-744-5p overexpression impact on EMT process in DU145 and LNCAP, qRT-PCR and western blot were employed to quantify expression of several EMT-related proteins (Fig. 3e and additional file 1: Fig. S1E), and according to the results, miR-744-5p mimics resulted in inhibited EMT process. Therefore, upregulated miR-744-5p repressed the cell growth of prostate cancer.

### UBE2L3 is the target gene of miR-744-5p

**Fig. 4:**
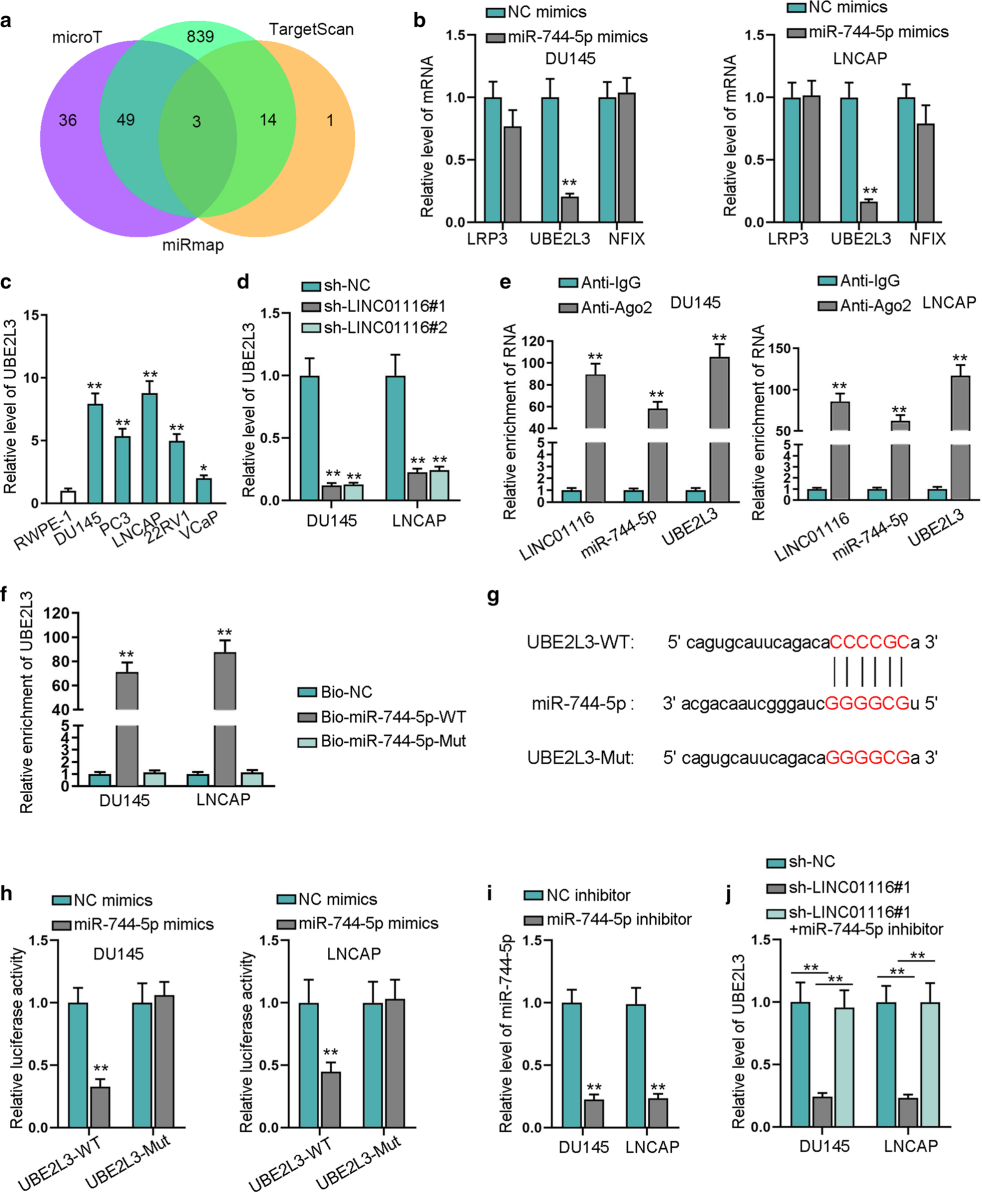
UBE2L3 is the target gene of miR-744-5p. **a** Three common mRNAs were screened out in microT, TargetScan, miRmap databases via ENCORI. **b** qRT-PCR was executed to quantify expression of the three mRNAs when miR-744-5p was overexpressed in cells. **c** qRT-PCR quantified the expression of UBE2L3 in prostate cancer cells and RWPE-1. **d** qRT-PCR tested the expression of UBE2L3 when the expression of LINC01116 was silenced in cells. **e**, **f** RIP and RNA pull down assays were utilized to prove the interaction of LINC01116, miR-744-5p and UBE2L3. **g** The alignment between UBE2L3 and miR-744-5p were predicted by ENCORI. **h** The luciferase activity of UBE2L3-WT was detected through luciferase reporter assay with miR-744-5p overexpression. **i** qRT-PCR detected the effectiveness of miR-744-5p inhibitor. **j** qRT-PCR was executed to quantify the expression of UBE2L3 when sh-LINC01116#1 or sh-LINC01116#1 + miR-744-5p inhibitor was transfected into cells. *P < 0.05, **P < 0.01

For the investigation of the potential mechanism of LINC01116, we searched for the downstream gene of miR-744-5p. We screened out three common messenger RNAs (mRNAs) of microT, TargetScan, and miRmap databases via ENCORI (Fig. 4a). And based on qRT-PCR results, after overexpressing miR-744-5p, the expression level of UBE2L3 was inhibited in cancer cells but no significant changes were presented in LRP3 and NFIX expression (Fig. 4b). So we took UBE2L3 as the experimental mRNA, from qRT-PCR, and UBE2L3 was discovered to be abnormally upregulated in prostate cancer cells (Fig. 4c). We predicted that UBE2L3 could be regulated by LINC01116, so we silenced the expression of LINC01116 and found that UBE2L3 expression was inhibited (Fig. 4d). For further verification, we conducted RIP to prove their correlation and the outcomes manifested LINC01116, miR-744-5p, UBE2L3 were enriched in Ago2 group, suggesting that they coexisted in the RISC (Fig. 4e). After the transfection of Bio-miR-744-5p-WT and Bio-miR-744-5p-Mut, it was found that UBE2L3 was enriched by Bio-miR-744-5p-WT (Fig. 4f). The alignment of UBE2L3 and miR-744-5p was found out through ENCORI (Fig. 4g). Then it was manifested through luciferase reporter assay that the fluorescence of UBE2L3-WT was dramatically reduced by miR-744-5p overexpression (Fig. 4h). Later, the effectiveness of miR-744-5p inhibitor was analyzed by qRT-PCR (Fig. 4i). Then the rescue assay was carried out, and based on the results, silencing LINC01116 led to the depletion of UBE2L3, and the inhibitory effect could be offset by co-transfection of miR-744-5p inhibitor into cells (Fig. 4j). So LINC01116 could regulate UBE2L3 through binding to miR-744-5p.

### UBE2L3 accelerates cell proliferation, migration and invasion of prostate cancer

**Fig. 5:**
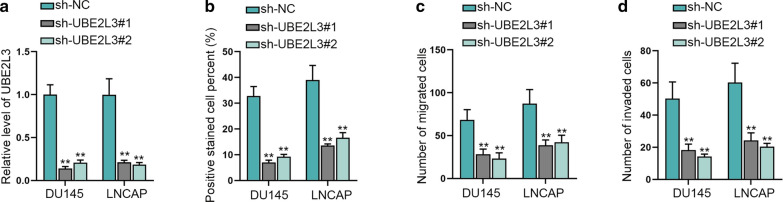
UBE2L3 accelerates cell proliferation, migration and invasion of prostate cancer. **a** qRT-PCR tested the inhibition efficiency of sh-UBE2L3. **b** EdU assay measured cell proliferative capability when UBE2L3 was inhibited in cells. **c**, **d** Transwell assay was utilized to estimate cell migratory and invasive capabilities after inhibiting UBE2L3 in cells. **P < 0.01

First, the effectiveness of sh-UBE2L3 was determined by implementation of qRT-PCR, and an obvious decrease in UBE2L3 expression was presented after transfection of sh-UBE2L3 into DU145 and LNCAP (Fig. 5a). Then we used EdU to detected cell proliferation and found that repressed UBE2L3 could weaken the proliferation capacity of cancer cells (Fig. 5b). Then we detected the quantity of successfully migratory or invaded cells via transwell assay and the results proved that after transfection with sh-UBE2L3, the quantity of migratory and invaded cells declined (Fig. 5c and d). Thus, it was confirmed that UBE2L3 accelerated prostate cancer cell proliferative, migratory and invasive capabilities.

### LINC01116 promotes growth of prostate cancer cells by regulating miR-744-5p/UBE2L3 axis

**Fig. 6:**
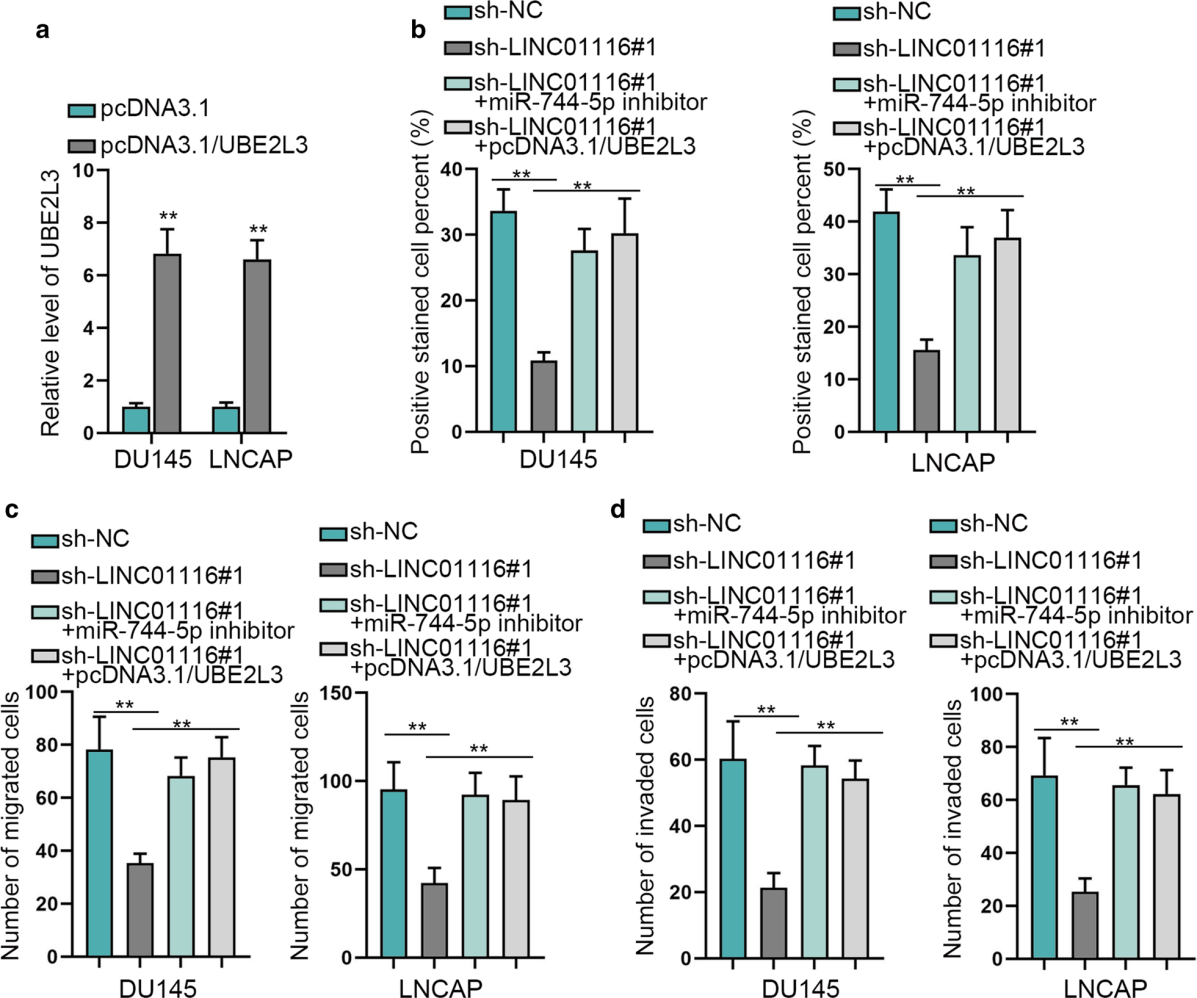
LINC01116 promotes growth of prostate cancer cells by regulating miR-744-5p/UBE2L3 axis. **a** Overexpression efficiency of pcDNA3.1/UBE2L3 was measured through qRT-PCR. **b** EdU assay detected cell proliferation in different groups. **c**, **d**. Transwell assay was utilized to estimate cell migratory and invasive capabilities in different groups. **P < 0.01

For the sake of investigation into the regulatory mechanism of LINC01116 in prostate cancer cells, with miR-744-5p and UBE2L3 involved, we conducted rescue assays. First of all, we assessed the effectiveness of pcDNA3.1/UBE2L3 in DU145 and LNCAP cells and qRT-PCR analysis displayed that the expression of UBE2L3 was upregulated by the transfection of pcDNA3.1/UBE2L3 (Fig. 6a). Then, in DU145 and LNCAP cells, it was discovered through EdU assay that silencing LINC01116 repressed cell proliferation and the inhibitory impact caused by sh-LINC01116#1 could be countervailed by co-transfection with miR-744-5p inhibitor or pcDNA3.1/UBE2L3 (Fig. 6b). The following transwell assays proved that inhibiting LINC01116 could decrease the number of migrated and invaded cancer cells, but co-transfection with miR-744-5p inhibitor or pcDNA3.1/UBE2L3 could counteract the repressive impact of silencing LINC01116 on cell migratory and invasive capabilities (Fig. 6c and d). Therefore, LINC01116 could promote cell growth of prostate cancer by regulating miR-744-5p/UBE2L3 axis.

## Discussion

Prostate cancer, an epithelial malignant tumor, features with a high occurrence rate globally and seriously affects people’s health. Recently, a large amount of study has focused on functions or mechanisms of lncRNAs in regulating cancer progression because lncRNAs can regulate the characteristics of assorted cancers [13], including prostate cancer. For example, overexpressed ANRIL accelerated the cell proliferation of prostate cancer via modulating let-7a/TGF-β1/Smad signaling pathway [14]. BRE-AS1 was reported to interact with miR-145-5p to regulate proliferation and apoptosis of prostate cancer [15]. And it was also reported that LOXL1-AS1 expedited the development of prostate cancer through miR-541-3p and CCND1 [16]. LINC01116 has been identified as a carcinogenic molecule for a variety of human cancers, including gastric cancer [17], osteosarcoma [18], glioma [19]. But no current studies have been carried out on its involvement in prostate cancer. The oncogenic function of LINC01116 in prostate cancer in this paper is the first time to be reported and the outcomes proved that LINC01116 was able to accelerate cell proliferation, migration, invasion and EMT process of prostate cancer.

Several previous studies have shown that there are many mechanisms by which lncRNA regulated gene expression [20, 21], and one of the important mechanisms is the interaction between lncRNAs and miRNAs [22]. CeRNA mechanism is a valuable mechanism of RNA interaction and has been discovered in different cancers. It refers to that lncRNA releases the shackles of mRNAs by sponging miRNAs at posttranscriptional level [23]. In the present study, we discovered that the location of LINC01116 was most in cytoplasm of prostate cancer cells, indicating that LINC01116 might act as a ceRNA at posttranscriptional level. Accordingly, we found and proved that miR-744-5p could be sponged by LINC01116 through bioinformatics tools and mechanism experiments. Moreover, previous studies have proved that knockdown of miR-744-5p could inhibit the growth and development of non-small cell lung cancer cells [24], ovarian cancer [25], and has an active role in the proliferation of lung adenocarcinoma cells through being regulated by lncRNA MAFG-AS1 [26]. Similarly, we confirmed that cell proliferative, migratory, invasive abilities as well as EMT progress could be inhibited by overexpressed miR-744-5p. Through bioinformatics tools, we found UBE2L3 could be targeted by miR-744-5p. The role of UBE2L3 has been investigated in many diseases. Our study also manifested UBE2L3 presented a high expression in prostate cancer cells, and overexpression of UBE2L3 also led to enhanced proliferative, migratory and invasive capabilities of prostate cancer cells.

## Conclusion

The present study showed that LINC01116 displayed a high level in prostate cancer cells, and could regulate miR-744-5p/UBE2L3 axis to promote cell growth of prostate cancer.

## Supplementary information

**Additional file 1: Figure S1.** A. Expression of E-cadherin and N-cadherin was quantified after LINC01116 knockdown. B. Expression of six EMT-related genes was measured via qRT-PCR when LINC01116 was inhibited. C. ENCORI database found the miRNAs (hsa-miR-744-5p, hsa-miR-650, hsa-miR-3612, hsa-miR-31-5p and hsa-miR-9-5p) that might bind to LINC01116. D. Expression levels of E-cadherin and N-cadherin in DU145 and LNCAP cells transfected with miR-744-5p mimics were quantified. E. Expression of six EMT markers was analyzed by qRT-PCR. **P < 0.01.

## Data Availability

Not applicable.

## Abbreviations

LINC01116: Long intergenic non-protein coding RNA 1116
UBE2L3: Ubiquitin conjugating enzyme E2 L3
lncRNA: Long noncoding RNA
mRNAs: Messenger RNAs
miRNAs: MicroRNAs
ceRNA: Competing endogenous RNA
PSA: Prostate specific antigen
MRI: Magnetic resonance imaging
circRNAs: Circular RNAs
ATCC: American Type Culture Collection
FBS: Fetal Bovine Serum
qRT-PCR: Quantitative real-time polymerase chain reaction
PFA: Paraformaldehyde
ECL: Enhanced chemiluminescence
EdU: 5-Ethynyl-2′-deoxyuridine
DAPI: 4′,6-diamidino-2-phenylindole
IF: Immunofluorescence
FISH: Fluorescence in situ hybridization
RIP: RNA binding protein immunoprecipitation
WT: wild-type
Mut: mutant
SD: standard deviation
ANOVA: analysis of variance

## References

[1] Srigley JR, Amin M, Boccon-Gibod L, Egevad L, Epstein JI, Humphrey PA, Mikuz G, Newling D, Nilsson S, Sakr W (2005). Prognostic and predictive factors in prostate cancer: historical perspectives and recent international consensus initiatives. Scand J Urol Nephrol Suppl.

[2] Stephenson AJ, Kattan MW, Eastham JA, Dotan ZA, Bianco FJ, Lilja H, Scardino PT (2006). Defining biochemical recurrence of prostate cancer after radical prostatectomy: a proposal for a standardized definition. J Clin Oncol.

[3] Barlow LJ, Badalato GM, Bashir T, Benson MC, McKiernan JM (2010). The relationship between age at time of surgery and risk of biochemical failure after radical prostatectomy. BJU Int.

[4] Guttman M, Rinn JL (2012). Modular regulatory principles of large non-coding RNAs. Nature.

[5] Gutschner T, Diederichs S (2012). The hallmarks of cancer: a long non-coding RNA point of view. RNA Biol.

[6] Sana J, Faltejskova P, Svoboda M, Slaby O (2012). Novel classes of non-coding RNAs and cancer. J Transl Med.

[7] Adelman K, Egan E (2017). Non-coding RNA: more uses for genomic junk. Nature.

[8] Anastasiadou E, Jacob LS, Slack FJ (2018). Non-coding RNA networks in cancer. Nat Rev Cancer.

[9] Ferreira HJ, Esteller M (2018). Non-coding RNAs, epigenetics, and cancer: tying it all together. Cancer metastasis reviews.

[10] Salmena L, Poliseno L, Tay Y, Kats L, Pandolfi PP (2011). A ceRNA hypothesis: the Rosetta Stone of a hidden RNA language?. Cell.

[11] Hu HB, Chen Q, Ding SQ (2018). LncRNA LINC01116 competes with miR-145 for the regulation of ESR1 expression in breast cancer. Eur Rev Med Pharmacol Sci.

[12] Fang YN, Huang ZL, Li H, Tan WB, Zhang QG, Wang L, Wu JL (2018). LINC01116 promotes the progression of epithelial ovarian cancer via regulating cell apoptosis. Eur Rev Med Pharmacol Sci.

[13] de Oliveira JC, Oliveira LC, Mathias C, Pedroso GA, Lemos DS, Salviano-Silva A, Jucoski TS, Lobo-Alves SC, Zambalde EP, Cipolla GA (2019). Long non-coding RNAs in cancer: another layer of complexity. J Gene Med.

[14] Zhao B, Lu YL, Yang Y, Hu LB, Bai Y, Li RQ, Zhang GY, Li J, Bi CW, Yang LB (2018). Overexpression of lncRNA ANRIL promoted the proliferation and migration of prostate cancer cells via regulating let-7a/TGF-β1/Smad signaling pathway. Cancer Biomark.

[15] Chen Z, Zhen M, Zhou J (2019). LncRNA BRE-AS1 interacts with miR-145-5p to regulate cancer cell proliferation and apoptosis in prostate carcinoma and has early diagnostic values. Biosci Rep.

[16] Long B, Li N, Xu XX, Li XX, Xu XJ, Liu JY, Wu ZH (2018). Long noncoding RNA LOXL1-AS1 regulates prostate cancer cell proliferation and cell cycle progression through miR-541-3p and CCND1. Biochem Biophys Res Commun.

[17] Su X, Zhang J, Luo X, Yang W, Liu Y, Liu Y, Shan Z (2019). LncRNA LINC01116 promotes cancer cell proliferation, migration and invasion in gastric cancer by positively interacting with lncRNA CASC11. OncoTargets Ther.

[18] Zhang B, Yu L, Han N, Hu Z, Wang S, Ding L, Jiang J (2018). LINC01116 targets miR-520a-3p and affects IL6R to promote the proliferation and migration of osteosarcoma cells through the Jak-stat signaling pathway. Biomed Pharmacother.

[19] Zhang N, Shuai K, Cheng J, Yang W, Kan Z (2019). LncRNA linc01116 prometes glioma cell migration and invasion by modulation of radixin targeted by miR-31. Int J Clin Exp Pathol.

[20] Yang L, Lin C, Jin C, Yang JC, Tanasa B, Li W, Merkurjev D, Ohgi KA, Meng D, Zhang J (2013). lncRNA-dependent mechanisms of androgen-receptor-regulated gene activation programs. Nature.

[21] Necsulea A, Soumillon M, Warnefors M, Liechti A, Daish T, Zeller U, Baker JC, Grutzner F, Kaessmann H (2014). The evolution of lncRNA repertoires and expression patterns in tetrapods. Nature.

[22] Paraskevopoulou MD, Hatzigeorgiou AG (2016). Analyzing MiRNA-LncRNA Interactions. Methods Mol Biol.

[23] Karreth FA, Pandolfi PP (2013). ceRNA cross-talk in cancer: when ce-bling rivalries go awry. Cancer Discov.

[24] Chen S, Shi F, Zhang W, Zhou Y, Huang J (2019). miR-744-5p inhibits non-small cell lung cancer proliferation and invasion by directly targeting PAX2. Technol Cancer Res Treat.

[25] Kleemann M, Schneider H, Unger K, Sander P, Schneider EM, Fischer-Posovszky P, Handrick R, Otte K (2018). MiR-744-5p inducing cell death by directly targeting HNRNPC and NFIX in ovarian cancer cells. Sci Rep.

[26] Sui Y, Lin G, Zheng Y, Huang W (2019). LncRNA MAFG-AS1 boosts the proliferation of lung adenocarcinoma cells via regulating miR-744-5p/MAFG axis. Eur J Pharmacol.

