# Supplementary figures and images for: Long non-coding RNA LINC01116 acts as an oncogene in prostate cancer cells through regulation of miR-744-5p/UBE2L3 axis
Source: Cancer Cell Int. 2021 Mar 16;21:168. doi: 10.1186/s12935-021-01843-w (PMC7962408; doi:10.1186/s12935-021-01843-w)

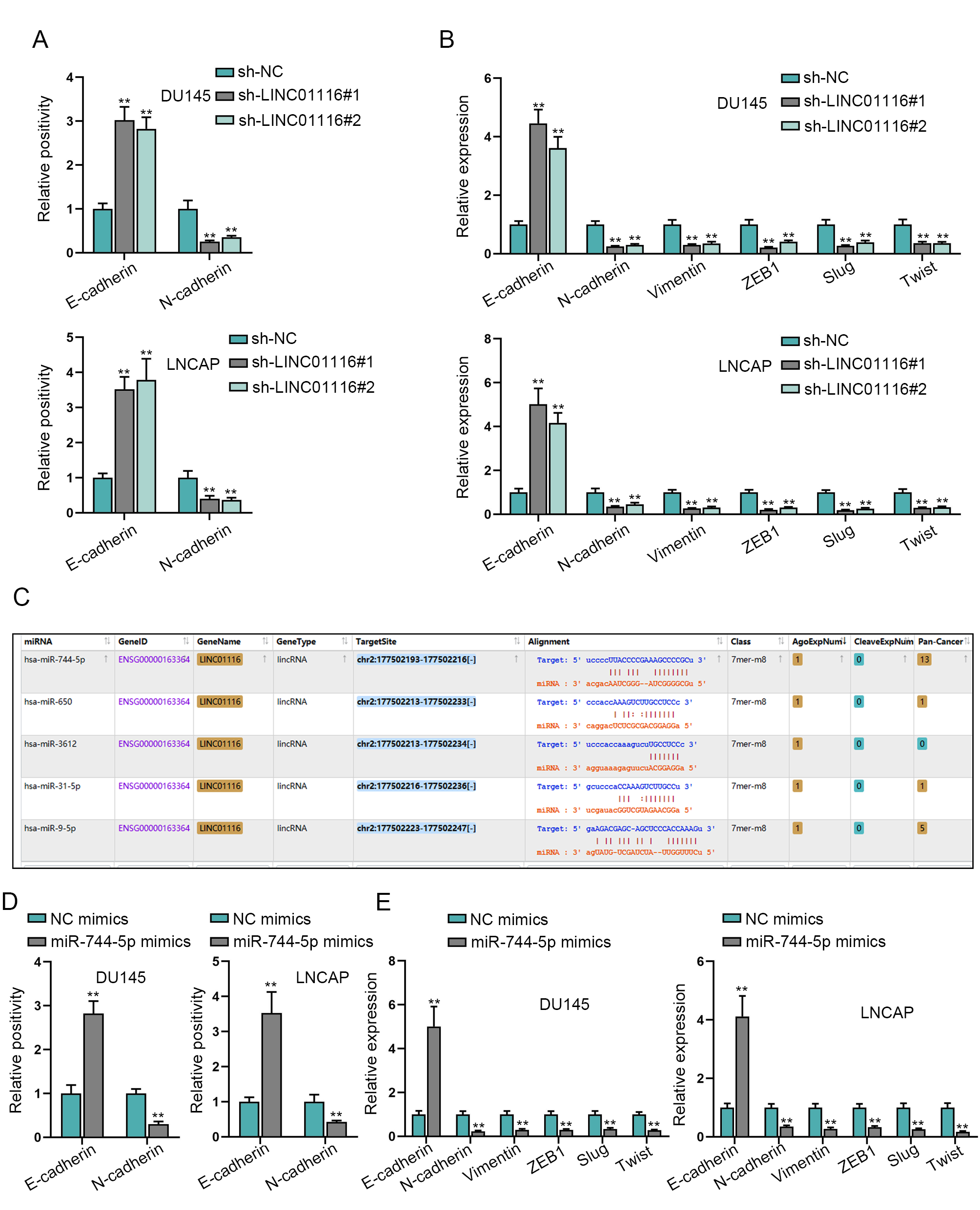

Supplement: Supplementary file 1 — Additional file 1: Figure S1. A. Expression of E-cadherin and N-cadherin was quantified after LINC01116 knockdown. B. Expression of six EMT-related genes was measured via qRT-PCR when LINC01116 was inhibited. C. ENCORI database found the miRNAs (hsa-miR-744-5p, hsa-miR-650, hsa-miR-3612, hsa-miR-31-5p and hsa-miR-9-5p) that might bind to LINC01116. D. Expression levels of E-cadherin and N-cadherin in DU145 and LNCAP cells transfected with miR-744-5p mimics were quantified. E. Expression of six EMT markers was analyzed by qRT-PCR. **P < 0.01. [file 12935_2021_1843_MOESM1_ESM.tif]
